# Anatomical, Physiological, Pathological, and Semeiological Researches on the Labial Glands

**Published:** 1843-01

**Authors:** 


ART. V. — *Recherches Anatomiques, Physiologiques, Pathologiques, et Séméiologiques, sur les Glandes Labiales.* Par A. A. SEBASTIAN, Professeur à l'Académie de Groningue.—*Groningue,* 1842. 4to, pp. 21. Avec une Planche.

*Anatomical, Physiological, Pathological, and Semeiological Researches on the Labial Glands.* By A. A. SEBASTIAN, Professor in the Academy of Groningen.—*Groningen,* 1842. With a Plate.

THE glands here treated of are generally known as forming a layer immediately beneath the mucous membrane of the lips. They have a conglomerate structure, with a single-trunked, branching duct, of which the branches seem to terminate in cells; and they secrete a transparent somewhat viscid fluid, containing elements and particles of epithelium, which the author supposes, though more from good reason than from evidence, to be analogous to saliva.

The novelty of the work is a somewhat detailed account of the diseases of these glands, which are as follows; and we may remark that we can confirm, in many respects, the account which is given.

1. *Obstruction of the excretory duct,* producing most commonly a small, transparent, vesicular, or hydatid-like tumour, which contains a clear viscid fluid. Such tumours are seldom larger than a pea, and are easily cured by puncture. More rarely the obstruction produces a small indolent, acne-like tumour or tubercle, which contains a thick, white, unctuous substance. 2. *Atrophy,* which the author has seen well marked in two cases of cancer of the lip. 3. *Active or passive congestion,* in which the glands are swollen and form small round or oval red eminences on the inner side of the lips. They secrete at the same time a large quantity of somewhat turbid or reddish fluid, with which even blood is sometimes mixed. This is the most frequent disease of the glands, and and it is especially common in young children, in whom it occurs chiefly, though not exclusively, in difficult dentition, in follicular duodenitis, and in typhoid fever. The author believes that the inflammation of these glands may be taken, with some confidence, as a sign of affection of the duodenum, for in all the fatal cases of typhus in which it was seen the duodenum was found to be diseased. 4. *Ulceration,* which is seen in the form of small deep ulcers; a rare disease and quite distinct from aphthæ and *muguet,* which the author has clearly determined have no connexion with the labial glands.

